# Supplementary material for: Duration of inter-pregnancy interval and its predictors among pregnant women in urban South Ethiopia: Cox gamma shared frailty modeling
Source: PLoS One. 2022 Aug 1;17(8):e0271967. doi: 10.1371/journal.pone.0271967 (PMC9342774; doi:10.1371/journal.pone.0271967)
Supplement: S1 Fig — (DOC) [file pone.0271967.s001.doc]

**S1 Fig. Kaplan-Meir survival graphs for the predictors of short IPI.**

1. **Age category**

1. **Education status**

1. **Modern contraceptive use**

1. **Duration of breast feeding**

1. **Survival status of child**

1. **Planning pregnancy**

1. **Discussion with husband**

1. **Husband encourage spacing**
