# Supplementary material for: Duration of inter-pregnancy interval and its predictors among pregnant women in urban South Ethiopia: Cox gamma shared frailty modeling
Source: PLoS One. 2022 Aug 1;17(8):e0271967. doi: 10.1371/journal.pone.0271967 (PMC9342774; doi:10.1371/journal.pone.0271967)
Supplement: S1 Table — (DOCX) [file pone.0271967.s002.docx]

**S1 Table. Log-rank test for the potential predictors (unadjusted) of short IPI.**

| Variables | P-values | Variables | P-values |
| --- | --- | --- | --- |
| Maternal age | 0.0206 | Counselled during PNC | 0.0001 |
| Maternal education status | 0.0483 | Exclusive breast feeding | 0.0001 |
| Greater number of children by sex | 0.0042 | Total duration of breast feeding | 0.0001 |
| Parity | 0.0352 | Decision maker for contraception | 0.0001 |
| Number of previous ANC visit | 0.0009 | Discussion with husband | 0.0001 |
| Recent number of children | 0.0085 | Husband encourages spacing | 0.0001 |
| Age at first childbirth | 0.4324 | Modern contraception | 0.0001 |
| Past history of stillbirth | 0.0088 | Plan to wait until current pregnancy | 0.0001 |
| Survival status of recent child | 0.0001 | Counselled during previous ANC visits | 0.0001 |
| Desired number of children | 0.4347 | Mode of delivery for recent child | 0.1824 |
| Wealth status | 0.0028 |  |  |
