# Supplementary material for: Duration of inter-pregnancy interval and its predictors among pregnant women in urban South Ethiopia: Cox gamma shared frailty modeling
Source: PLoS One. 2022 Aug 1;17(8):e0271967. doi: 10.1371/journal.pone.0271967 (PMC9342774; doi:10.1371/journal.pone.0271967)
Supplement: S1 Appendix — (DOCX) [file pone.0271967.s003.docx]

**S1 Appendix. R code to calculate the median hazard ratio (MHR)**

***# MHR for the Cox gamma frailty model***

A var.frailty denote the estimated variance of the frailty terms (which follow a gamma distribution).

df.F<-2/var.frailty

MHR<-qf(0.75,df.F,df.F)
