# Supplementary material for: Duration of inter-pregnancy interval and its predictors among pregnant women in urban South Ethiopia: Cox gamma shared frailty modeling
Source: PLoS One. 2022 Aug 1;17(8):e0271967. doi: 10.1371/journal.pone.0271967 (PMC9342774; doi:10.1371/journal.pone.0271967)
Supplement: Attachment — Submitted filename: PONE-D-21-12390_reviewer.pdf [file pone.0271967.s005.pdf]

PLOS ONE

Duration of inter-pregnancy interval and its predictors among pregnant women in urban South Ethiopia: Weibull inverse-Gaussian shared frailty modeling

--Manuscript Draft--

**Manuscript Number:**	PONE-D-21-12390
**Article Type:**	Research Article
**Full Title:**	Duration of inter-pregnancy interval and its predictors among pregnant women in urban South Ethiopia: Weibull inverse-Gaussian shared frailty modeling
**Short Title:**	Duration of inter-pregnancy interval and its predictors among pregnant women
**Corresponding Author:**	Belayneh Hamdela Jena, MPH<br>WCU: Wachemo University<br>Hossana, ETHIOPIA
**Keywords:**	Inter-pregnancy interval; pregnant women; urban South Ethiopia
**Abstract:**	Background<br><br>Short inter-pregnancy interval is a public health concern due to its adverse perinatal outcomes such as postpartum hemorrhage, anemia, premature birth, low birth weight, stillbirth and early neonatal death. Although understanding factors contributing to short inter-pregnancy interval is crucial to decrease the risk of these adverse outcomes, adequate evidence is lacking about the factors in urban context. Therefore, we aimed to assess duration of inter-pregnancy interval and its predictors among pregnant women in urban South Ethiopia.<br><br>Methods<br><br>A community-based retrospective follow up study was conducted among pregnant women in five geographically diverse urban settings in Hadiya zone, South Ethiopia. A total of 2171 pregnant women were included in the study at household level, from July 08/2019 to December 30/2019. A Weibull inverse-Gaussian shared frailty (random effect) model was applied for the analysis using STATA version 14 software. Adjusted hazard ratio (AHR) with 95% CI was used to assess statistically significant predictors at P<0.05.<br><br>Results<br><br>Median duration of inter-pregnancy interval was 22, 95%CI (21, 23), with inter-quartile range of 14 months. Having no formal education [AHR=0.65, 95%CI: 0.49, 0.84], contraceptive non-use [AHR=2.45, 95%CI: 2.10, 2.86], breast feeding for <24 months [AHR=5.97, 95%CI: 4.80, 7.41], death of recent child [AHR=2.49, 95%CI: 1.29, 4.82], plan pregnancy within 24 months [AHR=1.74, 95%CI: 1.29, 2.35], lack of discussion with husband about pregnancy spacing [AHR=1.29, 95%CI:1.07,1.55] and lack of husband encouragement about pregnancy spacing [AHR=1.19, 95%CI: 1.01, 1.42] were found to be predictors of duration of inter-pregnancy interval.<br><br>Conclusions<br><br>Duration of inter- pregnancy interval in the study settings was shorter than World Health Organization recommendation. There is a demand to improve contraceptive utilization and duration of breast feeding to optimize duration of inter-pregnancy interval. Men involvement in reproductive health services and advocating reproductive decision-making autonomy of women are fundamental.
**Order of Authors:**	Belayneh Hamdela Jena, MPH
	Gashaw Andargie Biks
	Yigzaw Kebede Gete
	Kassahun Alemu Gelaye

Powered by Editorial Manager® and ProduXion Manager® from Aries Systems Corporation

Additional Information:	
Question	Response
**Financial Disclosure**<br><br>Enter a financial disclosure statement that describes the sources of funding for the work included in this submission. Review the submission guidelines for detailed requirements. View published research articles from *PLOS ONE* for specific examples.<br><br>This statement is required for submission and **will appear in the published article** if the submission is accepted. Please make sure it is accurate.<br><br>**Unfunded studies**<br>Enter: *The author(s) received no specific funding for this work.*<br><br>**Funded studies**<br>Enter a statement with the following details:<br>• Initials of the authors who received each award<br>• Grant numbers awarded to each author<br>• The full name of each funder<br>• URL of each funder website<br>• Did the sponsors or funders play any role in the study design, data collection and analysis, decision to publish, or preparation of the manuscript?<br>• **NO** - Include this sentence at the end of your statement: *The funders had no role in study design, data collection and analysis, decision to publish, or preparation of the manuscript.*<br>• **YES** - Specify the role(s) played.<br><br>* typeset	The funders had no role in study design, data collection and analysis, decision to publish, or preparation of the manuscript.
**Competing Interests**<br><br>Use the instructions below to enter a competing interest statement for this submission. On behalf of all authors, disclose any competing interests that could be perceived to bias this work—acknowledging all financial support and any other relevant financial or non-financial competing interests.	The authors have declared that no competing interests exist.

This statement is required for submission and **will appear in the published article** if the submission is accepted. Please make sure it is accurate and that any funding sources listed in your Funding Information later in the submission form are also declared in your Financial Disclosure statement.<br><br>View published research articles from *PLOS ONE* for specific examples.<br><br>**NO authors have competing interests**<br><br>Enter: *The authors have declared that no competing interests exist.*<br><br>**Authors with competing interests**<br><br>Enter competing interest details beginning with this statement:<br><br>*I have read the journal's policy and the authors of this manuscript have the following competing interests: [insert competing interests here]*<br><br>* typeset	
**Ethics Statement**<br><br>Enter an ethics statement for this submission. This statement is required if the study involved:<br><br>• Human participants<br>• Human specimens or tissue<br>• Vertebrate animals or cephalopods<br>• Vertebrate embryos or tissues<br>• Field research<br><br>Write "N/A" if the submission does not require an ethics statement.<br><br>General guidance is provided below. Consult the submission guidelines for detailed instructions. **Make sure that all information entered here is included in the**	Ethical clearance was obtained from institutional review board (IRB) of University of Gondar, with registration number: O/V/P/RCS/05/1051/2019. Permission was obtained from regional and local health offices. Study participants were informed about how they were included in the study, the purpose of the study, their rights to withdraw or continue and potential benefits and harms of the study. Written consent form was prepared and attached together with questionnaire to obtain approval from each study participant by signature or fingerprint.

**Methods section of the manuscript.**<br><br>**Format for specific study types**<br><br>**Human Subject Research (involving human participants and/or tissue)**<br>• Give the name of the institutional review board or ethics committee that approved the study<br>• Include the approval number and/or a statement indicating approval of this research<br>• Indicate the form of consent obtained (written/oral) or the reason that consent was not obtained (e.g. the data were analyzed anonymously)<br><br>**Animal Research (involving vertebrate animals, embryos or tissues)**<br>• Provide the name of the Institutional Animal Care and Use Committee (IACUC) or other relevant ethics board that reviewed the study protocol, and indicate whether they approved this research or granted a formal waiver of ethical approval<br>• Include an approval number if one was obtained<br>• If the study involved *non-human primates*, add *additional details* about animal welfare and steps taken to ameliorate suffering<br>• If anesthesia, euthanasia, or any kind of animal sacrifice is part of the study, include briefly which substances and/or methods were applied<br><br>**Field Research**<br><br>Include the following details if this study involves the collection of plant, animal, or other materials from a natural setting:<br>• Field permit number<br>• Name of the institution or relevant body that granted permission	
**Data Availability**<br><br>Authors are required to make all data underlying the findings described fully available, without restriction, and from the time of publication. PLOS allows rare exceptions to address legal and ethical concerns. See the PLOS Data Policy and FAQ for detailed information.	Yes - all data are fully available without restriction

A Data Availability Statement describing where the data can be found is required at submission. Your answers to this question constitute the Data Availability Statement and **will be published in the article**, if accepted.<br><br>**Important:** Stating 'data available on request from the author' is not sufficient. If your data are only available upon request, select 'No' for the first question and explain your exceptional situation in the text box.<br><br>Do the authors confirm that all data underlying the findings described in their manuscript are fully available without restriction?	
**Describe where the data may be found in full sentences. If you are copying our sample text, replace any instances of XXX with the appropriate details.**<br><br>• If the data are **held or will be held in a public repository**, include URLs, accession numbers or DOIs. If this information will only be available after acceptance, indicate this by ticking the box below. For example: *All XXX files are available from the XXX database (accession number(s) XXX, XXX.).*<br>• If the data are all contained **within the manuscript and/or Supporting Information files**, enter the following: *All relevant data are within the manuscript and its Supporting Information files.*<br>• If neither of these applies but you are able to provide **details of access elsewhere**, with or without limitations, please do so. For example:<br><br>*Data cannot be shared publicly because of [XXX]. Data are available from the XXX Institutional Data Access / Ethics Committee (contact via XXX) for researchers who meet the criteria for access to confidential data.*<br><br>*The data underlying the results*	Data underlying the results will be available from the corresponding author via e-mail

presented in the study are available from (include the name of the third party and contact information or URL).<br>• This text is appropriate if the data are owned by a third party and authors do not have permission to share the data.<br><br>* typeset	
Additional data availability information:	

Powered by Editorial Manager® and ProduXion Manager® from Aries Systems Corporation

Manuscript

Click here to access/download;Manuscript;Manuscript on IPI Shared frailty analysis.doc

Duration of inter-pregnancy interval and its predictors among pregnant women in urban South Ethiopia: Weibull inverse-Gaussian shared frailty modeling

Belayneh Hamdela Jena[1,3*], Gashaw Andargie Biks[2], Yigzaw Kebede Gete[3], Kassahun Alemu Gelaye[3]

Affiliations determinants

[1]Department of Public Health, College of Medicine and Health Sciences, Wachemo University, Hossana, Ethiopia. E-mail: bhamdela@gmail.com

[2]Department of Health System and Policy, Institute of Public Health, College of Medicine and Health Sciences, University of Gondar, Gondar, Ethiopia. E-mail: gashawab@gmail.com

[3]Department of Epidemiology and Biostatistics, Institute of Public Health, College of Medicine and Health Sciences, University of Gondar, Gondar, Ethiopia. E-mail: kassalemu@gmail.com and gkyigzaw@yahoo.com

Corresponding author: bhamdela@gmail.com

Abstract

Background: Short inter-pregnancy interval is a public health concern due to its adverse perinatal outcomes such as postpartum hemorrhage, anemia, premature birth, low birth weight, stillbirth and early neonatal death. Although understanding factors contributing to short inter-pregnancy interval is crucial to decrease the risk of these adverse outcomes, adequate evidence is lacking about the factors in urban context. Therefore, we aimed to assess duration of inter-pregnancy interval and its predictors among pregnant women in urban South Ethiopia.

Methods: A community-based retrospective follow up study was conducted among pregnant women in five geographically diverse urban settings in Hadiya zone, South Ethiopia. A total of 2171 pregnant women were included in the study at household level, from July 08/2019 to December 30/2019. A Weibull inverse-Gaussian shared frailty (random effect) model was applied for the analysis using STATA version 14 software. Adjusted hazard ratio (AHR) with 95% CI was used to assess statistically significant predictors at $P<0.05$.

Results: Median duration of inter-pregnancy interval was 22, 95%CI (21, 23), with inter-quartile range of 14 months. Having no formal education [AHR=0.65, 95%CI: 0.49, 0.84], contraceptive non-use [AHR=2.45, 95%CI: 2.10, 2.86], breast feeding for <24 months [AHR=5.97, 95%CI: 4.80, 7.41], death of recent child [AHR=2.49, 95%CI: 1.29, 4.82], plan pregnancy within 24 months [AHR=1.74, 95%CI: 1.29, 2.35], lack of discussion with husband about pregnancy spacing [AHR=1.29, 95%CI:1.07,1.55] and lack of husband encouragement about pregnancy spacing [AHR=1.19, 95%CI: 1.01, 1.42] were found to be predictors of duration of inter-pregnancy interval.

Conclusions: Duration of inter- pregnancy interval in the study settings was shorter than World Health Organization recommendation. There is a demand to improve contraceptive utilization and duration of breast feeding to optimize duration of inter-pregnancy interval. Men involvement in reproductive health services and advocating reproductive decision-making autonomy of women are fundamental.

Key words: Inter-pregnancy interval; pregnant women; urban South Ethiopia

Introduction

Inter-pregnancy interval (IPI), also known as birth to pregnancy interval, is defined as the time elapsed from a live birth to subsequent conception or woman's last menstrual period (LMP) [1]. World Health Organization (WHO) recommended at least 24 months from a live birth to subsequent conception, and IPI less than 24 months is generally regarded as short interval [1], [2].

Every day in 2017, about 810 maternal deaths occur globally, 94% occurring in low and middle income countries [3]. Ethiopia, with a Maternal Mortality Ratio (MMR) of 412 per 100,000 live births, is one of the country in sub-Saharan Africa, contributing to unacceptably high (295,000) maternal death globally [3], [4]. Women in developing countries, including Ethiopia, have many more pregnancies and fertility rate than developed countries [5]. Pregnancies that are too many, too short and too long are public health concerns and catch the attention of policy and programs due to the adverse outcomes related with them [1]. To be specific, both too short and too long IPIs are closely linked to adverse maternal and neonatal outcomes [1], [6]. For instance, IPI <6, <18 and <24 months are associated with increased risk of prematurity, low birth weight and small for gestational age, which are linked to neonatal death [7], [8], [9]. IPI <18 months is linked to increased risk of adverse maternal outcomes such as anemia, postpartum hemorrhage and pre-eclampsia, that are further related

to maternal death [7], [10]. Longer IPI (>59 months) is related to pre-eclampsia [11]. Adequate IPI boosts maternal and child survival by decreasing these risks.

In Ethiopia, in contrary to the recommendation, sizeable proportion of women were not spacing pregnancies adequately. Still more than half of second and higher pregnancies occur within short interval than recommended [4]. More specifically, about 47.9% of women in urban areas have short IPI <24 months [2]. As many of developing countries, total fertility rate (TFR) in Ethiopia decline steadily [5] and it has taken 20 years to decline from 5.5 in 2000 and 4.2 in 2019; in average one child per woman [12]. The current TFR of 4.2 is still high and no more difference from the overall TFR in Sub-Saharan Africa (4.6) [5]. High TFR and short IPI could pose suffering and poor maternal health conditions in the country.

Previous studies identified factors such as age [13], maternal education [13], [14], [15], residence [2], wealth index [2], [14], sex of index child [14], perinatal death [15], breast feeding [2], [14], contraception [2], [14] and parity [13] to be associated with short birth intervals. However, it is noted that IPI can be affected by complex factors such as socioeconomic, demographic, political, cultural, population and health services [16]. To our knowledge, despite access to services, factors contributing to short IPI in urban settings is unclear. This study was proposed to fill this knowledge gap by considering the effect of clustering using shared frailty modelling approach, from five geographically diverse urban settings. Therefore, this study was aimed to assess duration of inter-pregnancy interval and its predictors among pregnant women in urban South Ethiopia. The findings would help to strengthen interventions targeted to optimize inter-pregnancy interval such as family planning programs. It will also contribute to accelerate maternal and child health related sustainable development goals.

Methods

Study setting

This study was conducted in Hadiya zone which is located in Southern Nations, Nationalities and Peoples Region (SNNPR), Ethiopia. The administrative center of the zone is Hossana town. In Hadiya zone there are one general hospital, three primary hospitals, 62 health Centers and 311 health posts that offer health services for the community [Hadiya Zone Health Bureau report-Unpublished]. In this study, five urban settings (Hossana, Shone, Gimbichu, Jajura and Homecho) which consist a total of eighteen kebeles (lowest administrative unit in Ethiopia) were included.

Population

A community-based retrospective follow up study was conducted among pregnant women who had a live birth during the most recent child birth from July 1/2014 onwards in five urban settings in Hadiya zone, South Ethiopia. All kebeles belonging to each urban were included in the study. All women who fulfil the following eligibility criteria were included: have given at least a live birth during the most recent child birth, have no recent abortion, have no recent stillbirth, recall the date of recent child birth or at least show immunization card, and pregnant during data collection. A total of 2171 pregnant women were identified through house-to-house identification from July 08/2019 to December 30/2019 and were included in the study. Data was collected by ten trained midwives and supervised by five public health professionals after two days training was given on the concepts of the questionnaire. Face to face interview was applied to collect retrospective data using structured questionnaire.

Measurements

The outcome variable was duration of inter-pregnancy interval. Inter-pregnancy interval was calculated by subtracting date of the most recent child birth from the date of last menstrual period. When women unable to recall date of last menstrual period, gestational age was estimated by using Ultrasound and then gestational age was subtracted from the date that a woman had the Ultrasound examination to obtain date of LMP. Duration of months from a live birth to conception or women's LMP was considered as a follow up period. Event (failure) was defined as the occurrence of pregnancy after a live birth within 24 months (short duration of IPI) while censored (success) was non-occurrence of pregnancy within 24 months (optimal duration of IPI) [1]. Time variable was duration of months spend from live birth to subsequent pregnancy or woman's LMP. The independent variables included in this study were socio-demographic and economic such as religion, ethnicity, marital status, age, sex of children, number of children, education, occupation and wealth index, and reproductive such as parity, age at first child birth, mode of delivery and breast feeding, and health service related such as contraceptive use, antenatal and postnatal care visits, counselling, place of delivery, and decision making related such as decision making for contraceptive use, discussion with husband about pregnancy spacing and whether the husband encourages wife to space pregnancies **(Table 1).**

Table 1. List of variables, definitions and measurements for the study in urban South Ethiopia, 2019.

**Variables**	**Measurements**
Inter-pregnancy interval (IPI)	Time duration from date of live birth to date of woman's last menstrual period in months categorized as event if pregnancy occurred in <24 months (short duration of IPI) or censored if it

	occurred at ≥24 months (optimal duration of IPI), based on World Health Organization recommendation for pregnancy spacing
Maternal age	Reported age of woman at interview in completed year categorized as 20-24, 25-29 and ≥30 years
Maternal education status	Education level of woman categorized as no formal, primary, secondary and higher education
Age at 1st child birth	Reported age at which a woman had the first child birth categorized as 15-19 and ≥20 years
Greater number of children by sex	Whether the family has more female, male or equal number of children by sex categorized as female, male and equal
Parity	Number of times a woman has given birth, irrespective of outcomes reported as 1, 2, 3 and ≥4
Number of previous ANC visits	Number of antenatal care visits a woman made during the preceding pregnancy categorized as <4 and ≥4, according to Ethiopia Demographic and Health Surveys (EDHS)
Recent number of children	Total number of children the family recently has categorized as 0-1, 2-3 and ≥4
Past history of Stillbirth	Whether a woman had history of giving a baby which has no sign of life such as no breathing, no heart beat and no movement before the most recent delivery responded as 'yes' if present 'no' otherwise

Survival status of the recent child	Whether the most recent child alive or died answered as alive or died
Counselled during previous ANC visits	Whether the woman received advice from health care providers about spacing pregnancies during here previous antenatal visits responded as yes or no
Counselled during PNC	Whether the woman received advice from health care providers about spacing pregnancies during postnatal period, including child immunization visits responded as yes or no
Exclusive breast feeding	If mother had given only breast milk to her recent child for up to six months, without any additional food or fluids, except medications categorized as yes if <6 or no if ≥ 6 months.
Total duration of breast feeding	Women were asked the total duration of breast feeding for the recent child until it was stopped in months, and then reported months categorized as <24 and ≥24
Decision maker on contraception	Whether a wife, husband or jointly they made decision to use contraception when it is needed reported as wife alone, husband alone and jointly (both)
Discussion with husband	If wife has made discussion or talk with her husband about spacing pregnancies after recent child birth responded as yes or no
Husband encourages spacing	Whether a husband encourages a wife to space pregnancies via safe methods of contraception when she requested and/or himself advise her to use responsibly responded as yes or no
Modern	If woman used any form of modern contraception after recent child

contraceptive use	birth responded as yes or no
Plan to wait until current pregnancy	Refers to for how long couples planned (if they have planned) to wait after recent child birth up to current pregnancy in months categorized as <24 and ≥24

Wealth index was measured using household assets for urban residence which consists of the following items: owner of house, number of rooms, material of roof, material of floor, material of exterior wall, source of drinking water, type of latrine, type of cooking materials (1=electricity, 0=wood/charcoal/biogas/natural gas, etc.), source of income, and presence or absence of; cell phone, refrigerator, radio, television, stove, chair, table, watch, modern bed, bicycle, Bajaj, motor cycle, car, donkey/horse cart and bank account. Each item was categorised in to two (1=yes and 0=No). Latrine and water sources were categorized as improved and unimproved facility based on world food program and WHO recommendations [17]. Principal component analysis was done to generate the components. Finally, ranking was done in to five categories (lowest, second, middle, fourth and highest).

Analysis

Data were entered using Epi-data version 3.1 and analyzed in STATA version 14 software. Descriptive statistics such as mean, median, standard deviations were done for continuous variables. Frequencies and percentages were calculated for categorical variables. For missed data, a complete case analysis was done. IPI is a time to event (from live birth to pregnancy) variable so that survival analysis model was fitted. Kaplan-Meir or product limit estimator was used to estimate cumulative survival probabilities and compare survival for predictors. Log-rank test was used to see if the graphs are significantly different for predictors of IPI. Cox-proportional hazard model was fitted to identify predictors of duration of IPI. Cox-

proportional hazard assumption was checked for the predictors by using global Shenfield residual test, and the assumption was violated (P=0.00001) (**Table 2**).

Table 2. Test of cox-proportional hazard assumption for the predictors of duration of inter-pregnancy interval in urban South Ethiopia, 2019.

**Variables**	**rho**	**Chi2**	**df**	**Prob>chi2**
Maternal age	0.00924	0.08	1	0.7809
Parity	0.05499	2.96	1	0.0852
Decision making on contraception	-0.00638	0.04	1	0.8471
Maternal education	0.04086	1.75	1	0.1853
Age at 1st child birth	-0.03983	1.52	1	0.2171
Greater number of children by sex	0.00431	0.02	1	0.8982
Modern contraceptive use	-0.14365	18.42	1	0.00001
Exclusive breast feeding	-0.03233	0.96	1	0.3267
Plan to wait until pregnancy	-0.00332	0.01	1	0.9202
Number of recent children	0.06915	4.60	1	0.0320
Husband encouragement	0.02790	0.75	1	0.3859
Discussion with husband	-0.01470	0.21	1	0.6460
Duration of breast feeding	-0.10567	9.78	1	0.0018
Counseled during postnatal care	0.04723	1.98	1	0.1593
Counseled during previous ANC	0.03759	1.29	1	0.2568
Number of previous ANC visits	0.00350	0.01	1	0.9141
Survival status of recent child	-0.07392	4.94	1	0.0262
Past history of stillbirth	0.03455	1.10	1	0.2932
**Global test**		**61.15**	**18**	**0.00001**

This we choose to fit parametric survival models. Since the data was obtained from five urban settings with a total of eighteen kebeles (clusters), checking whether there is unobserved heterogeneity due to clustering or not is crucial. This was checked by fitting a null model using parametric distributions (Exponential, Gompertz and Weibull), frailty distributions (gamma and inverse-Gaussian) and kebele as shared frailty (clustering) variable. A model with better fit was selected by using Log-likelihood, Akaike Information Criteria (AIC) and Bayesian Information Criteria (BIC). Weibull inverse-Gaussian shared frailty model (null model) provided higher Log-likelihood (-2320.7456), lower AIC (4647.491) and BIC (4664.54), and selected as better fit model for our data. The null model frailty parameter theta (θ=0.0885004, 95% CI: 0.0384386, 0.2037618) (LR test of theta=0: X^2 = 61.97, $P<0.001$) indicate that there is unobserved heterogeneity or shared frailty, as theta value and its 95%CI are greater than zero **(S1).** That means women from the same cluster are more or less prone to have short duration of IPI, and assuming dependency (correlation) within cluster would give more reliable estimates of the predictors. In bivariable Weibull inverse-Gaussian shared frailty model, variables that have shown statistically significant association with IPI at $P \leq 0.05$ were included in multivariable model. In multivariable Weibull inverse-Gaussian shared frailty model, adjusting for clustering and predictors, variables that have shown statistically significant association at $P<0.05$ and 95% CI for adjusted hazard ratio that did not include 1 were reported as predictors of duration of IPI. Interaction for possible effect modification was checked in the adjusted model. The results were interpreted using hazard ratio as an effect measure.

Quality control measures

The questionnaire was developed in English from related literature and Ethiopia Demographic and Health Survey (EDHS), and translated to Amharic language, and pre-tested

in similar setting (Durame town). Two days training was given for data collectors and supervisors on concept and approaching the participants. Supervisors checked data collection process closely. To minimize recall bias, we limited date of recent child birth in the last five years to help women in recalling data of last child birth. Family members such as husband, grandparent and mother-in-law were also involved to recall date of child birth. To minimize selection bias, all pregnant women during the study period were included using predefined eligibility criteria. Additionally, Ultrasound was used for those women who had difficulty of remembering date of LMP due to different reasons such contraceptive use and breast feeding. Epi-data was used to control errors during data entry. Data were explored to check outliers and missing values. Clustering effect was considered by fitting parametric shared frailty model and adjusted to control confounding variables to obtain reliable estimates of the predictors. Interaction was checked in the adjusted model for possible effect modifications.

Ethical clearance

Ethical clearance was obtained from institutional review board (IRB) of University of Gondar, with registration number: O/V/P/RCS/05/1051/2019. Permission was obtained from regional and local health offices. Study participants were informed about how they were included in the study, the purpose of the study, their rights to withdraw or continue and potential benefits and harms of the study. Written consent form was prepared and attached together with questionnaire to obtain approval from each study participant by signature or fingerprint.

Results

A total of 2202 pregnant women who fulfil the inclusion criteria in the five urban settings were identified. Of these, 31 women were refused to participate. This gives a response rate of

98.6%. The remained 2171 pregnant women were included in the study. The mean age of the study participants was 27.31 $\pm$ 3.44 years. Age of the participants range from 20-40 years. Majority, 1142 (54.5%) belongs to the age group of 25-29 years. Among the participants, 1936(89.3%) were Hadiya in ethnicity **(Table 3).**

Table 3. Socio-demographic and reproductive characteristics of study participants in five urban settings in Hadiya zone, South Ethiopia, 2019.

Variables	Frequency (%)
**Religion**	
Protestant	1898 (87.4)
Orthodox	115 (5.3)
Catholic	95 (4.4)
Muslim	52 (2.4)
Apostolic	11 (0.5)
**Ethnicity**	
Hadiya	1936 (89.2)
Kembata/Tembaro	114 (5.3)
Guragie/Siltie/Amhara/Oromo	121 (5.5)
**Marital status**	
Married	2133 (98.2)
Unmarried/ Divorced	38(1.8)
**Woman's main occupation**	
Housewife	1613 (74.3)
Employed	339 (15.7)
Merchant/ farmer/daily laborer/waiter	219 (10)

**Husband's main occupation**	
Daily laborer	599 (27.6)
Merchant	554 (25.5)
Employed	663 (30.6)
Farmer	274 (12.6)
Driver	81 (3.7)
**Husband's education**	
No formal education	315 (14.6)
Primary school (1-8 grades)	830 (38.4)
Secondary school (9-12 grades)	487 (22.5)
Higher education	532 (24.5)
**Wealth index**	
Lowest	430(20)
Second	435(20.2)
Middle	426(19.7)
Fourth	433(20.1)
Highest	431(20)
**Gravidity**	
2-3	1462 (67.4)
≥ 4	709 (32.6)
**Desired number of children**	
1-5	611 (28.1)
≥ 6	1198(55.2)
Undecided	362 (16.7)
**Place of delivery for recent child**	

Home	369 (17)
Health facility	1802 (83)
**Mode of delivery for recent child irth**	
Spontaneous vaginal delivery	2001 (92.2)
Cesarean section	109 (5)
Instrumental (Forceps and Vacuum)	61 (2.8)

Duration of inter-pregnancy interval

The median duration of IPI was 22, 95%CI (21, 23) months, with inter-quartile range of 14 months. During a follow up, a total of 1199 (55.2%) pregnancies occur within short interval (<24months). The cumulative probability of survival (remaining not pregnant) was decreasing as months after live birth increase **(Fig 1).**

Fig 1. Cumulative survival time to pregnancy in completed months among study participants in urban South Ethiopia, 2019.

Predictors of duration of inter-pregnancy interval

The log-rank test reported that maternal age, maternal education, parity, greater number of children by sex, recent number of children, number of previous ANC visits, past history of stillbirth, survival status of recent child, counseling during ANC, counseling during PNC, exclusive breast feeding, total duration of breast feeding, decision making for contraception, discussion with husband, husband encouragement for spacing, modern contraceptive use and planning pregnancy were significantly different for the probability of getting pregnancy after live birth at $P<0.05$ **(Table 4)**.

Table 4. Log-rank test for equality of survival for the predictors of duration of IPI in urban South Ethiopia, 2019.

Variables	P-values	Variables	P-values
Maternal age	0.0206	Counselled during PNC	0.0001
Maternal education status	0.0483	Exclusive breast feeding	0.0001
Greater number of children by sex	0.0042	Total duration of breast feeding	0.0001
Parity	0.0352	Decision maker for contraception	0.0001
Number of previous ANC visit	0.0009	Discussion with husband	0.0001
Recent number of children	0.0085	Husband encourages spacing	0.0001
Past history of stillbirth	0.0088	Modern contraception	0.0001
Survival status of recent child	0.0001	Plan to wait until current pregnancy	0.0001
Counselled during previous ANC	0.0001		

In multivariable Weibull inverse-Gaussian shared frailty model, having no formal education, contraceptive non-use, short duration of breast feeding, death of recent child, planning pregnancy for <24 months, not discussing with husband about pregnancy spacing and husband not encouraging pregnancy spacing were found to be statistically significant predictors of duration of IPI.

Women who have no formal education were 35% [AHR=0.65, 95%CI: 0.49, 0.84] less likely to have pregnancy within short duration after live birth than those who have higher education. Women who did not use any kind of modern contraceptive methods after recent child birth were 2.45 times [AHR=2.45, 95%CI: 2.10, 2.86] more likely to become pregnant shortly after child birth than contraceptive users. Women who discontinued breast feeding for the recent child within 24 months were nearly 6 times [AHR= 5.97, 95%CI: 4.80, 7.41] more likely to become pregnant within short duration as compared to those who continued breast feeding for the recommended duration (≥24 months). Women who lost their recent child (in the first month of life) were 2.49 times [AHR=2.49, 95%CI: 1.29, 4.82] more likely to have

pregnancy within short duration than women whose child alive. Women who had a plan to become pregnant within 24 months after a live birth were nearly 2 times [AHR=1.91, 95%CI: 1.41, 2.58] more likely to have pregnancy within short duration than women who planned for 24 and more months. Women who had no discussion with husband about pregnancy spacing were 29% [AHR=1.29, 95%CI: 1.07, 1.55] more likely to have pregnancy than their counter parts. Likewise, women whose husband did not encourage pregnancy spacing were 19% [AHR=1.19, 95%CI: 1.01, 1.42] more likely to have pregnancy within short duration after live birth than women whose husband encourages pregnancy spacing **(Table 5).**

Table 5. Multivariable Weibull inverse-Gaussian shared frailty survival model for the predictors of duration of inter-pregnancy interval among pregnant women in urban South Ethiopia, 2019.

**Variables**	**Pregnancy status**		**Crude HR+ (95% CI)**	**Adjusted HR++ (95% CI)**
	**Event (<24 months)**	**Censored (≥24 months)**		
**Maternal age**				
20-24	218 (60.6)	142 (39.4)	1	1
25-29	633 (55.4)	509 (44.6)	0.88 (0.75, 1.04)	0.95(0.78, 1.16)
≥30	307(51.9)	285(48.1)	0.78 (0.65, 0.94)**	0.86(0.66, 1.11)
**Maternal education status**				
No formal education	209(50.6)	204(49.4)	0.99(0.81, 1.22)	0.65(0.49, 0.84)**
Primary	515(55.9)	407(44.1)	1.18(0.99, 1.39)	0.91(0.75, 1.12)
Secondary	278(59.8)	187(40.2)	1.27(1.05, 1.53)*	1.07(0.88, 1.33)
Higher education	196(53.1)	173(46.9)	1	1

**Age at first child birth**				
15-19	283(54.7)	234(45.3)	1	1
≥20	890(55.3)	718(44.7)	1.23(1.06, 1.43)**	1.14(0.95, 1.37)
**Greater number of children by sex**				
Female	531(59.1)	365(40.9)	1	1
Male	460(54.1)	391(45.9)	0.88(0.78, 1.00)	0.98(0.84, 1.13)
Equal	205(49.5)	209(50.5)	0.74(0.63, 0.87)***	0.82(0.65, 1.02)
**Parity**				
1	511(58)	370(42)	1.34(1.13, 1.59)**	0.54(0.18, 1.57)
2	332(53.4)	290(46.6)	1.13(0.94, 1.36)	0.71(0.29, 1.74)
3	162(57)	122(43)	1.15(0.93, 1.41)	0.73(0.30, 1.77)
≥4	192(50.8)	185(49.2)	1	1
**Number of previous ANC visits**				
<4	479(59.4)	328(40.6)	1.26(1.09, 1.45)**	0.99(0.84, 1.18)
≥ 4	574(51.1)	549(48.9)	1	1
**Recent number of children**				
0-1	520(58.2)	374(41.8)	1	1
2-3	482(54.2)	407(45.8)	0.82(0.72, 0.93)**	0.80(0.44, 1.45)
≥4	187(50.5)	183(49.5)	0.73(0.61, 0.86)***	0.60(0.21, 1.74)
**Past history of Stillbirth**				
Yes	22(75.9)	7(24.1)	1.9(1.25, 2.90)***	1.19(0.69, 2.03)
No	1174(54.9)	964(45.1)	1	1
**Survival status of the recent child**				
Alive	1172(54.7)	970(45.3)	1	1
Died	25(92.6)	2(7.4)	6.52(4.36, 9.75)***	2.49(1.29, 4.82)**

**Counselled during previous ANC visits**				
Yes	790(50.6)	770(49.4)	1	1
No	401(67.6)	192(32.4)	1.70(1.49, 1.94)***	1.17(0.92, 1.47)
**Counselled during PNC visits**				
Yes	768(50.4)	756(49.6)	1	1
No	428(66.9)	212(33.1)	1.67(1.47, 1.90)***	0.99(0.78, 1.25)
**Exclusive breast feeding**				
Yes	1027(53.5)	892(46.5)	1	1
No	161(68.2)	75(31.8)	1.69(1.43, 2.01)***	0.98(0.69, 1.40)
**Duration of breast feeding**				
<24 months	1012(69)	455(31)	5.99(5.04, 7.11)***	5.97(4.80, 7.41)***
≥24 months	154(23.6)	499(76.4)	1	1
**Decision maker for contraception**				
Husband	305(66.9)	151(33.1)	1	1
Wife	92(53.5)	80(46.5)	0.69(0.54, 0.89)**	1.26(0.93, 1.72)
jointly	795(51.9)	738(48.1)	0.63(0.55, 0.73)***	1.07(0.88, 1.29)
**Discussion with husband**				
Yes	740(48.5)	786(51.5)	1	1
No	442(71.4)	177(28.6)	2.2(1.94, 2.49)***	1.29(1.07, 1.55)**
**Husband encourages spacing**				
Yes	767(50.4)	755(49.6)	1	1
No	412(68.1)	193(31.9)	1.78(1.57, 2.03)***	1.19(1.01, 1.42)*
**Modern contraceptive use**				
Yes	420(39.7)	639(60.3)	1	1
No	778(70)	333(30)	3.09(2.73, 3.49)***	2.45(2.10, 2.86)***

**Plan to wait until current pregnancy**				
<24 months	60(82.2)	13(17.8)	2.83(2.17, 3.68)***	1.91(1.41, 2.58)***
≥24 months	1035(54.2)	876(45.8)	1	1
**Exclusive breast feeding* Contraception**				
No contraception*no exclusive breast feeding				1.79(1.15, 2.78)*

Keys: *** = P<0.001, ** = P<0.01, * = P<0.05. + = adjusted for clustering, ++ = adjusted for predictors and clustering. HR: Hazard Ratio. CI: Confidence Interval. 1 = reference category.

Discussion

In this study, the median duration of IPI in the urban settings found to be short. Having no formal education was protective factor of short duration of IPI. Non-use of modern contraceptive methods, short duration of breast feeding, death of recent child, planning pregnancy within short duration, not making discussion with husband and husband not encouraging pregnancy spacing found to increase the chance of having pregnancy within short duration after birth.

Despite the attempts made to minimize, this study might have limitations. This study relayed on retrospective follow up data, it might have recall bias. Determining gestational age using Ultrasound and LMP might give different intervals in some extent, hence cumulative survival graph and survival probabilities need to be interpreted taking this in to consideration. Despite the limitations it will provide useful information for planning maternal health services.

The estimated median duration (22 months) of IPI in urban settings was lower than the estimates of Lemo, 24 months [2] and Dabat, 23.6 months [18] districts and national demographic and health survey, 25.5 months [4], Tanzania, 24.4 months [13] and

Bangladesh, 46 months [15]. The variations might be because of variation in estimating IPI by using LMP. We estimated IPI from date of LMP and Ultrasound result from pregnant women. For this discussion, IPI from those studies was estimated by subtracting 9 months of gestational age from birth interval. Population characteristics, study settings and sample size could be the other reasons. The desire for additional children might be vary in different settings even within country. Individual preferences for rearing children might vary; some people might wish to have children too closely so that they reach desired number of children within few years and then go soon to their business such as education and income generating activities to support their family. The estimated duration of IPI in this study is lower than WHO recommended duration of 24 months, suggesting presence of access to health services in the urban settings could not be guaranty for using modern contraception and spacing pregnancies [1].

In this study, women who had no formal education were less likely to have pregnancy within short duration after birth than women having higher education. Previous studies in Tanzania [13], Ethiopia [14] and Iran [19] have shown lower educated women had short birth interval. However, the finding is consistent with that of Bangladesh [15] and Korea [20], they reported women who completed higher education had an increased risk of short birth interval. This is not to mean women education has no importance rather higher educated women might have reasons related to their employment and further educational goals. Higher educated women might have better opportunity of employment, hence might breast feed less frequently due to lower contact time or lack of break, far away work place, fulltime employment and inflexible working time that contribute for fast return of fertility and increase risk of getting pregnancy [21], [22], [23]. On the other side, they might have further education plan so that they might stick to the schedule or plan and wish to have desired

number of children too closely in few years and give other breast milk substitutes instead of breast feeding for longer duration. Decision-making power of women with higher educational attainment due to economic independence on husband and ability to afford for breast milk substitutes and other ready-made infants food could be other possible reasons [24].

Women who did not use any form of modern contraceptive methods after recent child birth were more likely to have pregnancy within short duration than contraceptive users, and is consistent with others [2], [18], [19], [25], [26]. Contraception is the main tool to achieve optimal pregnancy interval, but can be affected by various factors that health service programs has to address, including myths and misconceptions (mainly fear of infertility) [27].

Women who breast feed recent child for shorter duration (<24 months) had pregnancy within short duration than those who breast feed for the recommended optimal duration (≥24 months). The finding is consistent with studies done in South Ethiopia [2], Bangladesh [15], Iran [19] and North Ethiopia [25]. This suggest breast feeding has positive impact on duration of IPI, and encouraging women to prolong breast feeding duration is advantageous. When a woman exclusively breast feed her child it can delay return of menses and promote pregnancy spacing [28], [29]. Extending breast feeding after exclusive breast feeding has positive contribution for spacing pregnancies in addition to increasing child survival. Although, frequency and duration of breast feeding can be influenced by personal, cultural, social, and environmental factors [30], in Ethiopia there is a good culture of breast feeding for longer duration that need to be promoted for further achievements in increasing pregnancy interval.

There was a positive interaction (agonistic) between modern contraception and exclusive breast feeding, when women who did not use modern contraceptive methods and at the same time who did not exclusively breast feed had higher chance of becoming pregnant shortly

after birth [AHR=1.79(1.15, 2.78)]. This indicate modern contraceptive use and exclusive breast feeding together are highly effective strategies for spacing pregnancies.

Women who had a plan to become pregnant within 24 months after recent child birth were more likely to have pregnancy in short duration than those planned for 24 and more months. This indicates that having just a plan might not be enough rather for how long is important in planning next pregnancy. Those who had scheduled their next pregnancy for longer duration might have their own reason. Thus, might have used contraception consistently to achieve their plan than those planned for shorter duration [26].

Women who did not discuss about pregnancy spacing following recent child birth have pregnancy within short duration than their counter parts. It is obvious that pregnancy and child birth need a joint decision of couples/relatives. Women who had a discussion with husband might have used postpartum contraception in order to space pregnancy [31], [32].

Likewise, women whose husband did not encourage pregnancy spacing were more likely to have pregnancy within short duration as compared to women whose husband encourages pregnancy spacing. This indicate that decision to space pregnancy fall on husband. Without getting approval from husband woman may not use any form of contraceptive methods to space pregnancies [32]. Preference for desired number of children might vary among couples and husband has more desire for additional children than women in Ethiopia [33]. It is also a common practice to see decisions regarding most of household issues and maternal health services made by husband. Husband involvement might be a crucial element of maternal health services including family planning methods [26], [33]. Considering the limitations aforementioned, the findings of this study could be generalized for urban settings in similar population.

Conclusions

Although maternal health services such as modern contraceptive and information could be accessed in urban settings, this study found that more than half of women in urban has short duration of IPI than World Health Organization recommendation. Short duration of IPI was related to modifiable factors like non-use of modern contraceptive methods, short duration of breast feeding, planning next pregnancy within short time and lack of decision-making autonomy of women to use maternal health services. Therefore, contraceptive utilization, duration of breast feeding and planning time of pregnancy has to be improved. Men involvement in reproductive health services and advocating reproductive decision-making autonomy of women are also fundamental, as decisions to use maternal health services like contraception are made usually by men. Women with higher education has to give emphasis for optimal pregnancy spacing. Contraceptive counselors need to emphasize on how to space, for how long and subsequent risk of inadequately spaced pregnancies.

Acknowledgment

We would like to thank University of Gondar and Wachemo University for financial support. We are very much thankful for study participants, data collectors and supervisors for their contributions.

References

1. World Health Organization. Report of a WHO technical consultation on birth spacing: Geneva, Switzerland 13-15 June 2005. World Health Organization; 2007. https://apps.who.int/iris/handle/10665/69855.
2. Yohannes S, Wondafrash M, Abera M, Girma E. Duration and determinants of birth interval among women of child bearing age in Southern Ethiopia. BMC pregnancy and childbirth. 2011 Dec;11(1):1-6. DOI: https://doi.org/10.1186/1471-2393-11-38.
3. World Health Organization. Trends in maternal mortality 2000 to 2017: estimates by WHO, UNICEF, UNFPA, World Bank Group and the United Nations Population Division. World Health Organization, 2019. https://apps.who.int/iris/handle/10665/327595.
4. Central Statistical Agency/CSA/Ethiopia and ICF. Ethiopia Demographic and Health Survey 2016, Addis Ababa, Ethiopia, and Rockville, Maryland, USA: CSA and ICF.
5. United Nations Department of Economic and Social Affairs, Population Division (2020). World Fertility and Family Planning 2020: Highlights (ST/ESA/SER.A/440).

https://www.un.org/en/development/desa/population/publications/pdf/family/World_Fertility_and_Family_Planning_2020_Highlights.pdf.
6. Conde-Agudelo A, Rosas-Bermúdez A, Kafury-Goeta AC. Birth spacing and risk of adverse perinatal outcomes: a meta-analysis. Jama. 2006 Apr 19;295(15):1809-23. DOI: 10.1001/jama.295.15.1809.
7. Lilungulu A, Matovelo D, Kihunrwa A, Gumodoka B. Spectrum of maternal and perinatal outcomes among parturient women with preceding short inter-pregnancy interval at Bugando Medical Centre, Tanzania. Maternal health, neonatology and perinatology. 2015 Dec;1(1):1-7. DOI: https://doi.org/10.1186/s40748-014-0002-1.
8. Grisaru-Granovsky S, Gordon ES, Haklai Z, Samueloff A, Schimmel MM. Effect of interpregnancy interval on adverse perinatal outcomes—a national study. Contraception. 2009 Dec 1;80(6):512-8. DOI: https://doi.org/10.1016/j.contraception.2009.06.006.
9. Smith GC, Pell JP, Dobbie R. Interpregnancy interval and risk of preterm birth and neonatal death: retrospective cohort study. Bmj. 2003 Aug 7;327(7410):313. DOI: https://doi.org/10.1136/bmj.327.7410.313.
10. Khan KS, Wojdyla D, Say L, Gülmezoglu AM, Van Look PF. WHO analysis of causes of maternal death: a systematic review. The lancet. 2006 Apr 1;367(9516):1066-74. DOI: https://doi.org/10.1016/S0140-6736(06)68397-9.
11. Conde-Agudelo A, Rosas-Bermúdez A, Kafury-Goeta AC. Effects of birth spacing on maternal health: a systematic review. American journal of obstetrics and gynecology. 2007 Apr 1;196(4):297-308. DOI: https://doi.org/10.1016/j.ajog.2006.05.055.
12. Ethiopian Public Health Institute (EPHI) [Ethiopia] and ICF. Ethiopia Mini Demographic and Health Survey 2019: Key Indicators. Rockville, Maryland, USA: EPHI and ICF.
13. Exavery A, Mrema S, Shamte A, Bietsch K, Mosha D, Mbaruku G, Masanja H. Levels and correlates of non-adherence to WHO recommended inter-birth intervals in Rufiji, Tanzania. BMC pregnancy and childbirth. 2012 Dec;12(1):1-8. DOI: https://doi.org/10.1186/1471-2393-12-152.
14. Hailu D, Gulte T. Determinants of short Interbirth interval among reproductive age mothers in Arba Minch District, Ethiopia. International journal of reproductive medicine. 2016 Oct;2016:17. DOI: https://doi.org/10.1155/2016/6072437.
15. De Jonge HC, Azad K, Seward N, Kuddus A, Shaha S, Beard J, Costello A, Houweling TA, Fottrell E. Determinants and consequences of short birth interval in rural Bangladesh: a cross-sectional study. BMC pregnancy and childbirth. 2014 Dec;14(1):1-7. DOI: https://doi.org/10.1186/s12884-014-0427-6.
16. Chen I, Jhangri GS, Lacasse M, Kumar M, Chandra S. Relationship between interpregnancy interval and adverse perinatal and neonatal outcomes in Northern Alberta. Journal of obstetrics and gynaecology Canada. 2015 Jul 1;37(7):598-605. DOI: https://doi.org/10.1016/S1701-2163(15)30197-3.
17. World Health Organization. Progress on sanitation and drinking-water 2013 update: Joint Monitoring Programme for Water Supply and Sanitation. URL: https://www.who.int/water_sanitation_health/publications/2013/jmp_report/en/.
18. Tessema GA, Zeleke BM, Ayele TA. Birth interval and its predictors among married women in Dabat District, Northwest Ethiopia: A retrospective follow up study. African journal of reproductive health. 2013 Jul 4;17(2):39-45. URL: https://www.ajol.info/index.php/ajrh/article/view/90265.
19. Fallahzadeh H, Farajpour Z, Emam Z. Duration and determinants of birth interval in Yazd, Iran: a population study. Iranian journal of reproductive medicine. 2013 May;11(5):379. URL: https://www.ncbi.nlm.nih.gov/pmc/articles/PMC3941411/.
20. Bumpass LL, Rindfuss RR, Palmore JA. Determinants of Korean birth intervals: the confrontation of theory and data. Population Studies. 1986 Nov 1;40(3):403-23. DOI: https://doi.org/10.1080/0032472031000142326.

21. Tadesse F, Alemayehu Y, Shine S, Asresahegn H, Tadesse T. Exclusive breastfeeding and maternal employment among mothers of infants from three to five months old in the Fafan zone, Somali regional state of Ethiopia: a comparative cross-sectional study. BMC Public Health. 2019 Dec;19(1):1-9. DOI: https://doi.org/10.1186/s12889-019-7345-5.
22. Ogbuanu C, Glover S, Probst J, Liu J, Hussey J. The effect of maternity leave length and time of return to work on breastfeeding. Pediatrics. 2011 Jun 1;127(6):e1414-27. DOI: https://doi.org/10.1542/peds.2010-0459
23. Kebede T, Woldemichael K, Jarso H, Bekele BB. Exclusive breastfeeding cessation and associated factors among employed mothers in Dukem town, Central Ethiopia. International breastfeeding journal. 2020 Dec 1;15(1):6. DOI: https://doi.org/10.1186/s13006-019-0250-9.
24. Yapo YV. Breastfeeding and child survival from 0 to 5 years in Côte d'Ivoire. Journal of Health, Population and Nutrition. 2020 Dec;39:1-9. DOI: https://doi.org/10.1186/s41043-020-0210-4.
25. Gebrehiwot SW, Abera G, Tesfay K, Tilahun W. Short birth interval and associated factors among women of child bearing age in northern Ethiopia, 2016. BMC women's health. 2019 Dec;19(1):1-9. DOI: https://doi.org/10.1186/s12905-019-0776-4.
26. Aleni M, Mbalinda SN, Muhindo R. Birth Intervals and Associated Factors among Women Attending Young Child Clinic in Yumbe Hospital, Uganda. International journal of reproductive medicine. 2020 Jan 4;2020. DOI: https://doi.org/10.1155/2020/1326596.
27. Ochako R, Mbondo M, Aloo S, Kaimenyi S, Thompson R, Temmerman M, Kays M. Barriers to modern contraceptive methods uptake among young women in Kenya: a qualitative study. BMC public health. 2015 Dec;15(1):1-9. DOI: https://doi.org/10.1186/s12889-015-1483-1
28. King J. Contraception and lactation. Journal of midwifery & women's health. 2007 Nov 1;52(6):614-20. DOI: https://doi.org/10.1016/j.jmwh.2007.08.012.
29. Brown RE. Breast-feeding and family planning: a review of the relationships between breast-feeding and family planning. The American journal of clinical nutrition. 1982 Jan 1;35(1):162-71. DOI: https://doi.org/10.1093/ajcn/35.1.162.
30. Kong SK, Lee DT. Factors influencing decision to breastfeed. Journal of advanced nursing. 2004 May;46(4):369-79. DOI: https://doi.org/10.1111/j.1365-2648.2004.03003.x.
31. Yonas Tadesse S, Emiru AA, Tafere TE, Asresie MB. Women's Autonomy Decision Making Power on Postpartum Modern Contraceptive Use and Associated Factors in North West Ethiopia. Advances in Public Health. 2019;2019. DOI: https://doi.org/10.1155/2019/1861570.
32. Tuloro T, Deressa W, Ali A, Davey G. The role of men in contraceptive use and fertility preference in Hossana Town, southern Ethiopia. Ethiopian Journal of Health Development. 2006;20(3). DOI: 10.4314/ejhd.v20i3.46826.
33. Tilahun T, Coene G, Temmerman M, Degomme O. Spousal discordance on fertility preference and its effect on contraceptive practice among married couples in Jimma zone, Ethiopia. Reproductive health. 2014 Dec;11(1):1-0. DOI: https://doi.org/10.1186/1742-4755-11-27.

Figure

Click here to access/download;Figure;Fig1.tif.tif

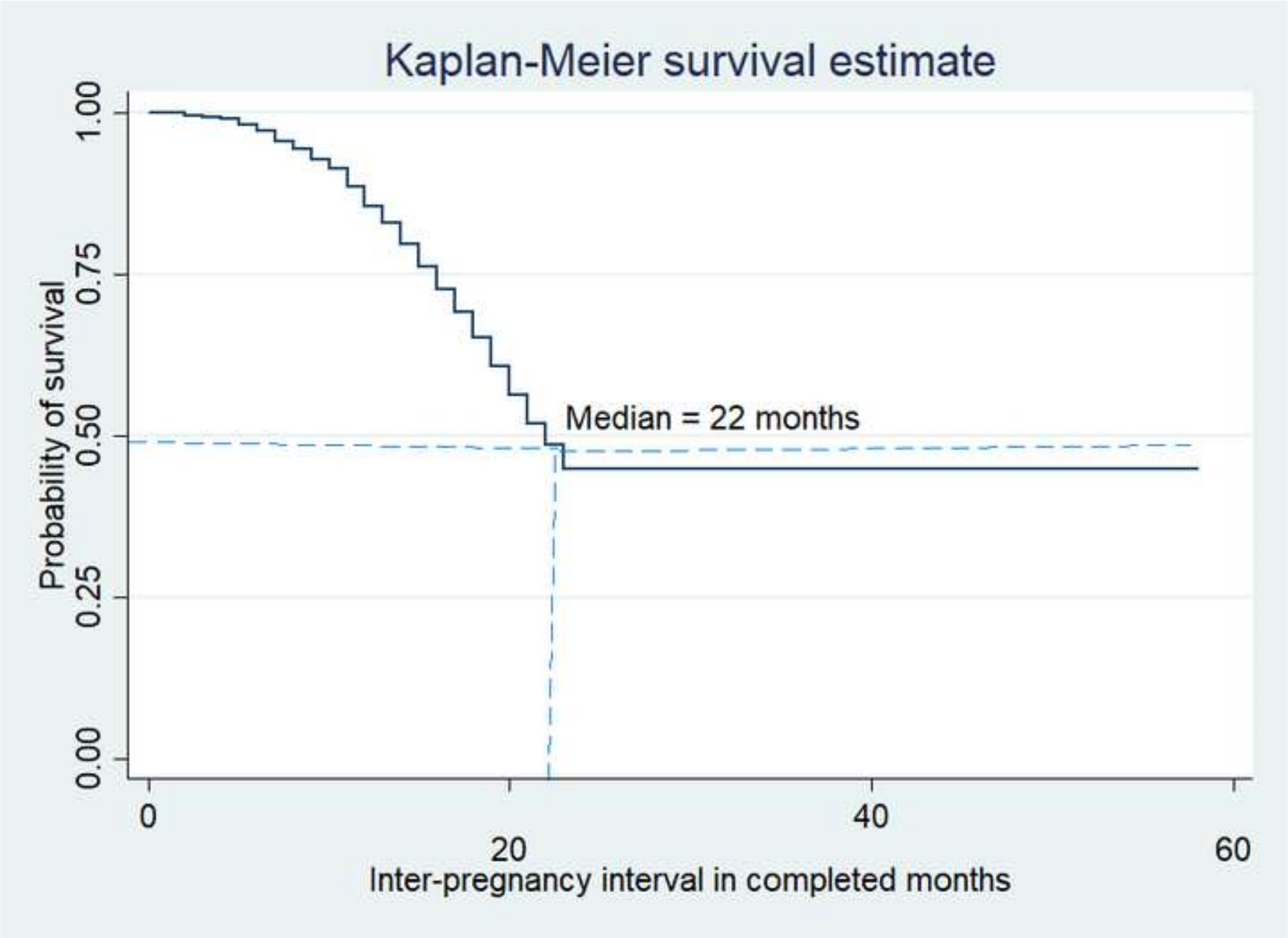

Click here to access/download
Supporting Information
S1. Model comparison (null model).doc
