# Supplementary material for: Duration of inter-pregnancy interval and its predictors among pregnant women in urban South Ethiopia: Cox gamma shared frailty modeling
Source: PLoS One. 2022 Aug 1;17(8):e0271967. doi: 10.1371/journal.pone.0271967 (PMC9342774; doi:10.1371/journal.pone.0271967)
Supplement: Attachment — Submitted filename: Dear Authors.docx [file pone.0271967.s007.docx]

Dear Authors thank you for your rigorous revision and addressing comments.

Some comments also

1. The finding of cluster effect needs to be added in the multivariable Cox gamma shared frailty model result table, in the discussion, abstract and conclusion as it has policy implication.
2. In the predictors section theta value better not more than three digits.
3. Simply base on full model variance of theta and its 95 CI calculate the median hazard with 95%CI and put it at the end of multivariable analysis and interpret it in short, then better to remove the cluster effect analysis section after table 4.

(MHR=1.30 (95CI%,1.11-1.43))
