# Supplementary material for: Duration of inter-pregnancy interval and its predictors among pregnant women in urban South Ethiopia: Cox gamma shared frailty modeling
Source: PLoS One. 2022 Aug 1;17(8):e0271967. doi: 10.1371/journal.pone.0271967 (PMC9342774; doi:10.1371/journal.pone.0271967)
Supplement: Attachment — Submitted filename: Response to Reviewers.docx [file pone.0271967.s008.docx]

**To:** **PLOS ONE** **Journal, Editorial Office**

**Subject:** Submitting a revised version of manuscript and response to **Reviewers.**

Ref: Submission ID PONE-D-21-12390R1

**Title of Article:** " Duration of inter-pregnancy interval and its predictors among pregnant women in urban South Ethiopia: Cox gamma shared frailty modeling"

**Authors:**

Belayneh Hamdela Jena, Gashaw Andargie Biks, Yigzaw Kebede Gete, Kassahun Alemu Gelaye

We would like to thank the Editor for facilitating and giving the opportunity to revise our manuscript. We are also grateful to reviewers for sharing their views and constructive comments. The comments are very important which will improve the quality of our manuscript. The point-by-point responses for each of the comments and the revised manuscript are provided in the attached documents.

**Regards,**

**The authors!**

**Editor’s comments:**

Journal Requirements:

Please review your reference list to ensure that it is complete and correct. If you have cited papers that have been retracted, please include the rationale for doing so in the manuscript text, or remove these references and replace them with relevant current references. Any changes to the reference list should be mentioned in the rebuttal letter that accompanies your revised manuscript. If you need to cite a retracted article, indicate the article’s retracted status in the References list and also include a citation and full reference for the retraction notice.

**Authors’ response:** Thank you dear Editor for your time and contribution. We checked the references and no retracted references found.

**Reviewers’ comments**

**Reviewer #1**

Some comments also
1. The finding of cluster effect needs to be added in the multivariable Cox gamma shared frailty model result table, in the discussion, abstract and conclusion as it has policy implication.

**Authors’ response:** Thank you dear reviewer for your contribution. We acknowledge your comments. In the revised manuscript we added the cluster effect in all sections according to your suggestion (kindly see the highlighted text in the table, discussion, abstract and conclusion sections of the revised manuscript).

2. In the predictors section theta value better not more than three digits.

**Authors’ response:** Thank you dear reviewer now we have corrected the digits into three

3. Simply base on full model variance of theta and its 95 CI calculate the median hazard with 95%CI and put it at the end of multivariable analysis and interpret it in short, then better to remove the cluster effect analysis section after table 4.
(MHR=1.30 (95CI%,1.11-1.43))

**Authors’ response:** Thank you dear reviewer we have calculated the MHR and its 95% CI from full model variance of theta, and put the interpretation in short, under the multivariable analysis. We have deleted the cluster effect analysis section as well.

**Reviewer #2:**

GENERAL: There are still several grammatical errors and wrong use of tenses that need to be corrected throughout the text.

**Authors’ response:** Thank you dear reviewer for your contribution. We acknowledge your comments. In the revised manuscript we have attempted to correct those errors throughout the text as much as possible.

ABSTRACT: The last sentence in the results sub-section is rather confusing and should be recasted.

**Authors’ response:** Thank you dear reviewer now we have corrected it. The MHR is normally interpreted in that manner.

ITRODUCTION: Page 4, lines 1-2,it is not clear what the authors mean by pregnancies that were too long or short. Are they referring to duration of pregnancy or inter-pregnancy interval? In page 4, paragraph 2,line 1, it is not clear which recommendation the authors are referring to and the recommendation should be referenced.

**Authors’ response:** Thank you dear reviewer for suggesting to correct those vague words. In the revised manuscript we have corrected them. Too long and too short were referring to inter-pregnancy intervals. We have also clarified which recommendation we were referring, and cited as well. It refers world health organization recommendation of at least 24 months.

METHODS: Under sample size and data collection- line 4-5, the calculation of inter-pregnancy interval seams to be different from that in lines 1-3 under variables and measurements. When and where were the face to face interviews conducted and how were the identified houses selected?

**Authors’ response:** Thank you dear reviewer for the comments. We approximated inter-pregnancy interval from birth interval estimate, by subtracting 9 months of average gestational age, which is possible when information for inter-pregnancy interval is lacking, just to estimate sample size. For instance, birth interval 33 months is approximately equivalent to inter-pregnancy interval 24 months (33-9 = 24). Birth interval is could be estimated by subtracting the date of preceding childbirth (delivery) from the date of the recent childbirth (birth interval = date of recent childbirth minus date of preceding childbirth). Inter-pregnancy interval = date of last menstrual period minus date of the recent childbirth. The difference between birth interval and inter-pregnancy interval is the ‘gestational age’. Inter-pregnancy interval estimation does not include gestational age. So inter-pregnancy interval could be estimated from birth interval by subtracting the gestational age when information about inter-pregnancy interval is lacking. This was done because we lacked an inter-pregnancy interval estimates to calculate the sample size during design of the study. However, for our study we have both dates (date of last childbirth and date of last menstrual period or estimate from Ultrasound) information, and the population were pregnant women. The gestational age of women was also known so it is possible to estimate inter-pregnancy interval without birth interval information.

We have done a house-to-house visits in the urban community, and the face-to-face interviews were conducted during the visits, at the house-hold level. For this study, we included all eligible pregnant women who are willing to participate, during the study period. No sampling of houses was done, rather we considered all eligible pregnant women who meet predefined inclusion criteria.

DISCUSSION: Several grammatical errors. Page 23, paragraph 2, the last sentence should be recasted.

**Authors’ response:** Thank you dear reviewer we attempted to correct the grammatical errors in all sections of the manuscript. We preferred to remove the sentence as it is non-sensing to discuss in that manner.

END!
